# Association Between Dietary Monounsaturated Fatty Acid Intake and Metabolic Syndrome Among Korean Adults: A Cross-Sectional Analysis of Korea National Health and Examination Survey

**DOI:** 10.3390/nu17101629

**Published:** 2025-05-09

**Authors:** Bo-Hyun Choi, Sunhye Shin

**Affiliations:** 1 Department of Pharmacology, School of Medicine, Daegu Catholic University, Daegu 42472, Republic of Korea; bchoi@cu.ac.kr; 2 Department of Food and Nutrition, Seoul Women’s University, Seoul 01797, Republic of Korea

**Keywords:** monounsaturated fatty acid, metabolic syndrome, dietary intake, hypertriglyceridemia, Korea National Health and Examination Survey

## Abstract

Introduction/Objectives: Although monounsaturated fatty acids (MUFAs) are known as a healthy nutrient, their impact on the risk of metabolic syndrome (MetS) in the Asian population is not fully understood. This study aimed to determine the association between dietary MUFA intake and the prevalence of MetS among Korean adults. Materials and Methods: The 7th Korea National Health and Examination Survey (2016–2018) was analyzed. MetS was defined based on the guideline of the National Cholesterol Education Program Adult Treatment Panel III (NCEP-ATP III) criteria, and MUFA intake was calculated using a single 24 h dietary recall. Data from 3932 younger adults (19–39 years), 6943 middle-aged adults (40–64 years), and 3942 older adults (≥65 years) were included and multivariable logistic regression models were applied to estimate odds ratios (OR) and 95% confidence intervals (CI). Results: Approximately 25.8% of Korean adults showed signs of MetS, and the average MUFA intake was 13.70 g/day. Middle-aged adults with a higher MUFA intake had a lower risk of MetS (OR 0.52, 95% CI 0.35–0.78 for men; OR 0.66, 95% CI 0.43–0.99 for women) compared to those with a lower MUFA intake after the adjustment of possible confounding variables, including age, body mass index, total energy intake, household income, alcohol consumption, smoking, aerobic exercise, and energy intake from carbohydrates. No significant associations were observed in younger and older adults. Conclusions: These results suggest that higher dietary MUFA consumption is associated with a lower risk of MetS in middle-aged Korean adults. These findings suggest that including MUFA-rich foods in the diet could be a practical strategy to reduce the burden of MetS in clinical and public heath settings.

## 1. Introduction

Metabolic syndrome (MetS) is a multifactorial condition characterized by the co-occurrence of abdominal obesity, hypertriglyceridemia, reduced high-density lipoprotein cholesterol (HDL-C), and elevated blood pressure, hyperglycemia, which collectively increases the risk of type 2 diabetes, cardiovascular diseases, and all-cause mortality [1,2]. Globally, the prevalence of MetS is estimated to be around 20–25% of the adult population, with significant variation depending on ethnicity, age, and lifestyle [3]. With the global rise in obesity and sedentary lifestyles, the prevalence of MetS has significantly increased across both developed and developing countries, posing a substantial public health challenge [4]. In Asian countries, including Korea, the prevalence of MetS has also been rising steadily, with recent national surveys indicating a prevalence rate of approximately 30% among Korean adults [4,5].

Dietary factors play a central role in the development and prevention of MetS. Recent research has highlighted the detrimental effects of diets high in saturated fats and refined carbohydrates [6]. In contrast, dietary intake of both *n*-3 and *n*-6 fatty acids is negatively associated with MetS prevalence, indicating diets rich in polyunsaturated fatty acids (PUFAs) exert beneficial effects [6,7]. Compared to PUFAs, monounsaturated fatty acids (MUFAs) are less susceptible to lipid peroxidation, which makes MUFAs exert more favorable effects on lipid profiles, insulin sensitivity, and inflammatory markers [8,9,10,11]. Predominantly found in olive oil, nuts, and avocados, MUFAs are a key component of the Mediterranean diet, which has been associated with reduced risk of metabolic and cardiovascular diseases [12,13].

Despite growing evidence from Western populations, limited research has examined the relationship between dietary MUFA intake and MetS in Asian populations whose dietary patterns and metabolic risk profiles differ. In Korea, MUFA intake is relatively low, and most dietary MUFAs are derived from animal-based sources, such as pork belly, rather than plant-based oils [14]. Koreans consume approximately 20 kg of pork belly per year [15], and 100 g of pork belly contains 14 g of MUFAs, including palmitoleic acid (16:1*n*-7; 0.8 g), vaccenic acid (18:1*n*-7; 0.9 g), and oleic acid (OLA; 18:1*n*-9; 12 g) [16]. Meanwhile, less than 25% of Korean adults consume nuts (rich in MUFAs), and the average amount of nut consumption of the consumers (12.7 g/day) is far below the recommendation (30.0 g/day) of multiple dietary guidelines [17]. In addition, Asians tend to have a higher percentage of body fat and increased visceral adiposity at lower BMI levels compared to Western populations, potentially modifying the impact of dietary components on metabolic health [18,19]. Given these differences, it remains unclear whether the protective effects of MUFAs observed in Western populations extend to Koreans.

To address this gap, we analyzed nationally representative data from the 7th Korea National Health and Nutrition Examination Survey (KNHANES VII 2016–2018) which includes comprehensive health, nutrition, and dietary assessments. The aim of this study was to evaluate the association between dietary MUFA intake and the prevalence of MetS among Korean adults. Since the impact of MUFA intake may vary according to age and sex due to differences in metabolic rates, hormonal profiles, and dietary patterns [19,20], the association was examined with stratification by age and sex. Understanding the impact of MUFA intake in the Korean context may provide valuable insights for dietary guidelines and public health strategies to reduce the burden of MetS in Asians.

## 2. Materials and Methods

### 2.1. Data Source and Subjects

The data analyzed in this study is the 7th Korea National Health and Nutrition Examination Survey (KNHANES VII 2016–2018). The KNHANES is a nationwide cross-sectional survey that is conducted every year by the Korea Centers for Disease Control and Prevention (KCDC). The target population is nationally representative non-institutionalized civilians ≥ 1 year in Korea, and the survey includes health interviews, health examinations, and dietary intake assessment. A new dataset of approximately 10,000 individuals is added each survey year [21]. All participants provided informed consent, and analyses of the data adhered to the Helsinki Declaration. The data collection for KNHANES VII was approved by the Institutional Review Board (IRB) of KCDC (IRB No. 2018-01-03-P-A), and the ethical review and approval were waived by the IRB of Seoul Women’s University for this study (IRB No. SWU IRB-2023A-02).

Of the 24,269 participants in the KNHANES VII, exclusions were sequentially made for children ≤ 18 years (*n* = 4880), individuals missing dietary intake data (*n* = 2535), those with extreme energy intake (<500 kcal/day or >4000 kcal/day; *n* = 613), and those without health examination data (*n* = 1424). Consequently, data from 14,817 participants were analyzed. To account for potential biological differences, participants were categorized by age and sex as follows: men 19–39 years (*n* = 1616), women 19–39 years (*n* = 2316), men 40–64 years (*n* = 2788), women 40–64 years (*n* = 4155), men ≥ 65 years (*n* = 1718), and women ≥ 65 years (*n* = 2224).

### 2.2. Health Interview

Via a self-administered health interview questionnaire, information on demographic details, socioeconomic status, personal behaviors, and medical conditions was collected. Household income was classified into quartiles: low, middle-low, middle-high, and high. For KNHANES VII-1 (2016), the cutoff values are KRW 750.0, 1500.0, and 2463.1 thousands. For KNHANES VII-2 (2017), the cutoff values are KRW 894.4, 1905.7, and 3104.2 thousands. For KNHANES VII-3 (2018), the cutoff values are KRW 1060.7, 2020.7, and 3179.6 thousands. Individuals who have drunk alcohol more than once a month in the preceding year were categorized as current alcohol consumers. Those who smoked over 100 cigarettes in their lifetime and continued smoking were labeled as current smokers. Regular aerobic exercise was defined as conducting more than 150 min of physical activity with moderate intensity, 75 min of physical activity with vigorous intensity, or an equivalent mixture of moderate and vigorous physical activity (when 1 min of vigorous physical activity is considered equal to 2 min of moderate activity) per week.

### 2.3. Health Examination

Body weight, height, waist circumference, blood pressure, and blood profiles were measured by trained medical personnel by using periodically calibrated equipment. The body mass index (BMI) was calculated as weight in kilograms divided by squared height in meters. According to the guideline of the National Cholesterol Education Program Adult Treatment Panel III (NCEP-ATP III), participants who met at least 3 of the following criteria were defined as at risk of MetS: (1) waist circumference (WC) ≥ 90 cm in men or ≥80 cm in women based on the Asian cutoffs, (2) triglycerides (TG) ≥ 150 mg/dL, (3) HDL-C < 40 mg/dL in men or <50 mg/dL in women, (4) blood pressure ≥ 130/85 mmHg or usage of blood pressure medication, (5) fasting blood glucose (FBG) ≥ 100 mg/dL, usage of glucose lowering medication, or insulin treatment.

### 2.4. Dietary Intake

Through the face-to-face interview method, trained dietitians collected a single 24 h dietary recall, assessing dietary consumption in the past 24 hours, from the participants. The daily intake of total energy, individual foods, and nutrients were calculated using the KNHANES recipe and food composition database published by the Korean Rural Development Administration [12]. For dietary MUFA intake, the processed data of nutrient intake provided by the KNHANES VII was utilized.

### 2.5. Statistical Analysis

General characteristics, divided by quartiles of dietary MUFA intake, were described using means ± standard errors (SE) for continuous variables or by presenting numbers with percentages for categorical variables. Differences in overall characteristics among the quartiles were demonstrated by Student’s *t*-test for continuous variables or by Rao–Scott chi-squared tests for categorical variables.

The risks of MetS based on dietary MUFA intake were assessed using multivariate logistic regression models with the lowest quartile of dietary MUFA intake (Q1) set as the reference group. The analyses incorporated four models: the unadjusted model estimated crude odds ratios (ORs) and 95% confidence intervals (CIs); Model 1 was adjusted for age, BMI, and total energy intake; Model 2 was additionally adjusted for household income, alcohol consumption, smoking habits, and regular aerobic exercise; and Model 3 additionally included energy intake from carbohydrates.

All statistical analyses were performed using SPSS software (Version 26, IBM, Armonk, NY, USA) based on the survey procedure [21]. A two-sided *p*-value < 0.05 was considered statistically significant.

## 3. Results

Supplementary Tables S1–S3 show the general characteristics of participants. The MetS prevalence in Korean adults ≥ 19 years was 25.8%. By age and sex, 15.1% among younger men 19–39 years, 6.8% of younger women 19–39 years, 33.8% of middle-aged men 40–64 years, 24.5% of middle-aged women 40–64 years, 33.6% of older men ≥ 65 years, and 54.5% of older women ≥ 65 years had MetS. The quartiles of dietary MUFA consumption were as follows: 10.31, 16.61, and 24.57 g/day for younger men; 7.74, 12.74, and 24.57 g/day for younger women; 6.95, 11.78, and 18.89 g/day for middle-aged men; 5.56, 9.35, and 14.99 g/day for middle-aged women; 3.70, 7.02, and 12.12 g/day for older men; and 2.43, 4.97, and 8.77 g/day for older women.

Although MUFA intake was not significantly associated with socioeconomic status or personal behaviors among younger adults 19–39 years (Supplementary Table S1), middle-aged adults 40–64 years and older adults ≥ 65 years with higher MUFA intake tended to be younger, more likely to have higher household income, be current alcohol consumers, and do more regular aerobic exercise (Supplementary Tables S2 and S3). Middle-aged men with a lower MUFA intake tended to have higher FBG and systolic blood pressure (SBP), and middle-aged women with a lower MUFA intake tended to have higher BMI, WC, TG, FBG, SBP, and diastolic blood pressure, and lower HDL-C (Supplementary Table S2). Older women with lower MUFA intake tended to have higher WC and TG, and lower HDL-C (Supplementary Table S3).

Dietary MUFA intake was shown to be associated with MetS among middle-aged adults 40–64 years. After adjusting confounding variables, middle-aged men in the highest quartile (Q4) of MUFA intake had 48% lower risk (OR, 0.52; 95% CI, 0.35−0.78), and middle-aged women in Q4 and the second highest quartile (Q3) had 34% (OR, 0.66; 95% CI, 0.43−0.99) and 30% lower risk (OR, 0.70; 95% CI, 0.50−0.98) of MetS compared to middle-aged men and women in the lowest quartile (Q1), respectively. Meanwhile, there was no association between MUFA intake and MetS among younger men 19–39 years and older men ≥ 65 years, and beneficial association between the two variables among younger women 19–39 years and older women ≥ 65 years disappeared with the adjustment of confounding factors (Table 1).

Among the five components of MetS, dietary MUFA intake was strongly associated with hypertriglyceridemia in middle-aged adults 40–64 years. With the adjustment for confounding variables, middle-aged men in Q2 (OR, 0.72; 95% CI, 0.54−0.95), Q3 (OR, 0.65; 95% CI, 0.49−0.87), and Q4 (OR, 0.56; 95% CI, 0.40−0.78), and middle-aged women in Q3 (OR, 0.71; 95% CI, 0.52−0.97) and Q4 (OR, 0.64; 95% CI, 0.43−0.96) had lower risk of hypertriglyceridemia compared to their counterparts in Q1 (Table 2). In addition, middle-aged men in Q4 (OR, 0.65; 95% CI, 0.47−0.90) and middle-aged women in Q3 (OR, 0.66; 95% CI, 0.50−0.88) had a lower risk of hyperglycemia compared to their counterparts in Q1 (Supplementary Tables S4–S7).

## 4. Discussion

In this large and nationally representative dataset with a cross-sectional design, we found that a higher dietary MUFA intake was significantly associated with a lower risk of MetS among middle-aged Korean adults aged 40–64 years. Specifically, middle-aged men in the highest quartile of MUFA intake had 48% lower odds of MetS compared to those in the lowest quartile, and middle-aged women in the highest and second highest quartiles also exhibited a significantly reduced risk. However, no such associations were observed in younger adults 19–39 years or older adults ≥ 65 years after adjusting potential confounding variables.

These findings align with previous research suggesting the beneficial effects of MUFA-rich diets, particularly in improving cardiometabolic health markers [8]. The Mediterranean diet, which emphasizes MUFA intake primarily from plant-based sources such as olive oil and nuts, has been shown to reduce the risk of MetS, cardiovascular diseases, and type 2 diabetes [12,13,22]. Although dietary MUFA are often derived from animal-based sources, such as pork and eggs, in Korea [14,23], our findings suggest that a higher total MUFA consumption, regardless of dietary sources, may contribute to improved metabolic profiles, particularly in middle-aged adults.

The observed age-specific associations may be partially explained by differences in metabolic vulnerability, lifestyle factors, and dietary behaviors across age groups. Middle-aged individuals tend to exhibit higher rates of insulin resistance, visceral adiposity, and elevated blood pressure, which may be more responsive to dietary modulation [24]. On the other hand, the absence of significant associations in younger and older adults could be due to the lower baseline MetS prevalence in young adults and the greater impact of comorbidities and age-related physiological changes in older adults. These factors may override the effects of dietary components.

Potential biological mechanisms underlying the protective effects of MUFA include reduction in oxidative stress and inflammation, improvements in lipid metabolism, and enhancement of insulin sensitivity [8,9,10,11]. Both experimental and clinical studies have demonstrated that MUFA improves metabolic fitness through anti-oxidative and anti-inflammatory pathways. MUFA was shown to promote fatty acid oxidation in the liver, skeletal muscle, and adipose tissues, which clears excessive fatty acids and reduces overnutrition-induced chronic inflammation [25,26,27], and this was related to lower serum triglycerides and blood pressure, and improved HDL-C levels and glycemic control [28,29,30]. Our results consistently present that middle-aged individuals 40–64 years with lower MUFA intake tended to have more adverse profiles in key components of MetS, such as elevated fasting blood glucose, triglycerides, and blood pressure, supporting the mechanistic insights.

### 4.1. Perspective for Clinical Practice

The findings of this study have important implications for clinical and community health practices aimed at preventing and managing metabolic syndrome and related chronic conditions. Particularly in middle-aged individuals at risk of metabolic abnormalities or type 2 diabetes, dietary counseling to promote MUFA-rich food intake could serve as a key component of lifestyle modification programs.

Given that the majority of Korean MUFA intake comes from animal-based sources, clinical dietitians and case manager nurses, who deliver tailored nutritional and behavioral support, should also guide patients toward incorporating more plant-based MUFA, such as nuts, avocados, and vegetable oils, to maximize health benefits. Such integrated and evidence-based care approaches would align with global standards in preventive cardiometabolic care [23,31]. A growing body of literature supports the effectiveness of lifestyle medicine case manager nurses for patients with obesity, metabolic syndrome, or type 2 diabetes. Nurse-led interventions have been shown to effectively reduce body weight, improve metabolic parameters, and enhance patient engagement in health-promoting behaviors [32,33,34]. Integrating the roles of clinical dietitians and case manager nurses into primary care could enhance early intervention for individuals at high risk.

From public health perspectives, our findings are particularly relevant given the increasing burden of MetS among Korean adults [4]. Middle-aged adults 40–64 years represent a critical window for intervention, where dietary modifications such as increasing MUFA intake could meaningfully reduce the disease risk. Although there was no association between MUFA intake and MetS risk among older adults ≥ 65 years in the current study, intake of MUFA-rich nuts was reported to negatively correlate with the risk of low muscle strength, one of the common diseases in elderly, in older Korean adults [17]. Therefore, public health messaging and dietary guidelines emphasizing fat quality, that is dietary fatty acid composition, may benefit Korean populations at risk of other chronic diseases in addition to MetS.

### 4.2. Limitations

Several strengths of this study should be noted, including the utilization of nationally representative data from KNHANES, comprehensive adjustment for demographic and lifestyle factors, and stratified analyses by age and sex. However, some limitations should also be considered. First, the cross-sectional design of KNHANES precludes causal inference; therefore, the observed association between MUFA intake and MetS should not be interpreted as causal. Second, dietary intake was assessed using a single 24 h recall, which may not capture habitual intake and is subject to recall bias. Third, we could not account for the source of MUFA (plant vs. animal-based), which may have differential health effects and influence the observed association.

## 5. Conclusions

Our findings suggest that higher dietary MUFA intake is associated with the lower prevalence of MetS among middle-aged Korean adults. These results underscore the importance of dietary fat composition in cardiometabolic health and support the potential value of MUFA-rich diets as a preventive strategy for MetS in Korean populations. From a clinical standpoint, incorporating MUFA-based dietary counseling into lifestyle medicine interventions—led by clinical dietitians and case manager nurses—may be a promising approach for managing MetS and preventing type 2 diabetes and cardiovascular diseases. Further longitudinal and intervention studies are warranted to confirm these associations and to clarify the impact of MUFA sources and mechanisms in the pathogenesis of MetS.

## Figures and Tables

**Table 1:** Risks of metabolic syndrome according to quartiles of dietary monounsaturated fatty acid intake among Korean adults by age and sex.

Monounsaturated Fatty Acid Intake	Unadjusted		Model 1 *		Model 2 †		Model 3 ‡	
	OR	95% CI	OR	95% CI	OR	95% CI	OR	95% CI
19–39 years								
Men								
Q1	1 (ref)		1 (ref)		1 (ref)		1 (ref)	
Q2	1.32	0.87, 1.99	1.33	0.81, 2.17	1.36	0.81, 2.29	1.34	0.79, 2.25
Q3	0.97	0.63, 1.50	0.90	0.52, 1.54	0.96	0.55, 1.67	0.93	0.53, 1.62
Q4	1.02	0.68, 1.54	0.77	0.42, 1.41	0.75	0.39, 1.45	0.70	0.36, 1.39
Women								
Q1	1 (ref)		1 (ref)		1 (ref)		1 (ref)	
Q2	0.91	0.57, 1.44	1.09	0.61, 1.95	1.08	0.59, 1.97	1.06	0.57, 1.98
Q3	0.93	0.59, 1.46	1.11	0.60, 2.08	1.10	0.58, 2.10	1.07	0.52, 2.21
Q4	0.48	0.29, 0.79	0.63	0.28, 1.45	0.60	0.25, 1.43	0.58	0.21, 1.58
40–64 years								
Men								
Q1	1 (ref)		1 (ref)		1 (ref)		1 (ref)	
Q2	0.97	0.76, 1.24	0.92	0.69, 1.22	0.99	0.73, 1.34	0.90	0.66, 1.23
Q3	1.02	0.79, 1.31	0.88	0.65, 1.20	0.96	0.69, 1.33	0.82	0.59, 1.15
Q4	0.74	0.57, 0.96	0.64	0.45, 0.91	0.69	0.47, 1.01	0.52	0.35, 0.78
Women								
Q1	1 (ref)		1 (ref)		1 (ref)		1 (ref)	
Q2	0.63	0.51, 0.79	0.78	0.61, 0.996	0.78	0.61, 1.01	0.80	0.61, 1.05
Q3	0.47	0.37, 0.59	0.62	0.47, 0.82	0.67	0.50, 0.89	0.70	0.50, 0.98
Q4	0.49	0.39, 0.61	0.58	0.43, 0.79	0.61	0.45, 0.84	0.66	0.43, 0.99
≥65 years								
Men								
Q1	1 (ref)		1 (ref)		1 (ref)		1 (ref)	
Q2	0.90	0.65, 1.24	1.00	0.68, 1.47	1.12	0.75, 1.67	1.10	0.73, 1.65
Q3	1.02	0.75, 1.40	1.17	0.80, 1.73	1.40	0.92, 2.12	1.35	0.88, 2.08
Q4	1.06	0.77, 1.45	1.24	0.81, 1.90	1.33	0.85, 2.08	1.25	0.77, 2.05
Women								
Q1	1 (ref)		1 (ref)		1 (ref)		1 (ref)	
Q2	0.86	0.64, 1.15	0.98	0.71, 1.36	1.02	0.73, 1.42	0.96	0.67, 1.36
Q3	0.63	0.47, 0.85	0.76	0.54, 1.06	0.79	0.56, 1.12	0.71	0.47, 1.05
Q4	0.59	0.43, 0.80	0.73	0.49, 1.08	0.81	0.54, 1.22	0.65	0.37, 1.13

* Model 1: adjusted for age, body mass index, and total energy intake. † Model 2: model 1 plus household income, alcohol consumption, smoking, and aerobic exercise. ‡ Model 3: model 2 plus energy from carbohydrates.

**Table 2:** Risks of hypertriglyceridemia ^1^ according to quartiles of dietary monounsaturated fatty acid intake among Korean adults by age and sex.

Monounsaturated Fatty Acid Intake	Unadjusted		Model 1 *		Model 2 †		Model 3 ‡	
	OR	95% CI	OR	95% CI	OR	95% CI	OR	95% CI
19–39 years								
Men								
Q1	1 (ref)		1 (ref)		1 (ref)		1 (ref)	
Q2	0.98	0.70, 1.37	0.91	0.63, 1.32	0.98	0.67, 1.43	0.92	0.63, 1.34
Q3	0.74	0.54, 1.01	0.66	0.46, 0.93	0.69	0.48, 0.99	0.61	0.42, 0.89
Q4	1.04	0.77, 1.41	0.88	0.60, 1.31	0.94	0.63, 1.42	0.74	0.48, 1.15
Women								
Q1	1 (ref)		1 (ref)		1 (ref)		1 (ref)	
Q2	0.88	0.59, 1.33	0.89	0.55, 1.42	0.91	0.55, 1.49	0.87	0.53, 1.43
Q3	0.86	0.58, 1.27	0.80	0.50, 1.27	0.82	0.50, 1.36	0.75	0.43, 1.31
Q4	0.62	0.41, 0.95	0.57	0.31, 1.05	0.55	0.28, 1.06	0.47	0.21, 1.04
40–64 years								
Men								
Q1	1 (ref)		1 (ref)		1 (ref)		1 (ref)	
Q2	0.82	0.65, 1.04	0.73	0.57, 0.94	0.76	0.58, 0.99	0.72	0.54, 0.95
Q3	0.83	0.66, 1.06	0.68	0.52, 0.89	0.71	0.53, 0.93	0.65	0.49, 0.87
Q4	0.76	0.60, 0.96	0.59	0.44, 0.79	0.64	0.47, 0.88	0.56	0.40, 0.78
Women								
Q1	1 (ref)		1 (ref)		1 (ref)		1 (ref)	
Q2	0.87	0.70, 1.07	0.99	0.79, 1.23	0.98	0.78, 1.24	0.98	0.77, 1.24
Q3	0.58	0.45, 0.74	0.68	0.52, 0.89	0.72	0.54, 0.94	0.71	0.52, 0.97
Q4	0.57	0.44, 0.72	0.63	0.46, 0.87	0.65	0.47, 0.89	0.64	0.43, 0.96
≥65 years								
Men								
Q1	1 (ref)		1 (ref)		1 (ref)		1 (ref)	
Q2	0.91	0.65, 1.28	0.92	0.65, 1.31	0.92	0.65, 1.32	0.88	0.61, 1.26
Q3	0.94	0.67, 1.31	0.93	0.65, 1.32	0.96	0.67, 1.37	0.88	0.61, 1.27
Q4	0.98	0.71, 1.35	0.37	0.65, 1.45	0.97	0.64, 1.46	0.82	0.52, 1.29
Women								
Q1	1 (ref)		1 (ref)		1 (ref)		1 (ref)	
Q2	0.85	0.64, 1.14	0.85	0.63, 1.16	0.89	0.65, 1.22	0.85	0.61, 1.19
Q3	0.79	0.59, 1.07	0.77	0.55, 1.07	0.77	0.55, 1.09	0.71	0.47, 1.07
Q4	0.69	0.52, 0.92	0.62	0.43, 0.88	0.66	0.46, 0.95	0.57	0.33, 0.97

^1^ Hypertriglyceridemia was defined as triglycerides ≥ 150 mg/dL. * Model 1: adjusted for age, body mass index, and total energy intake. † Model 2: model 1 plus household income, alcohol consumption, smoking, and aerobic exercise. ‡ Model 3: model 2 plus energy from carbohydrates.

## Data Availability

The data (KNHANES VII) presented in this study are available at https://knhanes.kdca.go.kr/knhanes/main.do (accessed on 6 May 2025).

## Supplementary Materials

The following supporting information can be downloaded at: https://www.mdpi.com/article/10.3390/nu17101629/s1, Table S1: General characteristics of the subjects aged 19–39 y according to dietary monounsaturated fatty acid intake; Table S2: General characteristics of the subjects aged 40–64 y according to dietary monounsaturated fatty acid intake; Table S3: General characteristics of the subjects aged ≥65 y according to dietary monounsaturated fatty acid intake; Table S4: Risks of abdominal obesity1 according to quartiles of dietary monounsaturated fatty acid intake among Korean adults by age and sex; Table S5: Risks of hypo-HDL-cholesterolemia1 according to quartiles of dietary monounsaturated fatty acid intake among Korean adults by age and sex; Table S6: Risks of hypertension1 according to quartiles of dietary monounsaturated fatty acid intake among Korean adults by age and sex; Table S7: Risks of hyperglycemia1 according to quartiles of dietary monounsaturated fatty acid intake among Korean adults by age and sex.
