# Supplementary material for: Association Between Dietary Monounsaturated Fatty Acid Intake and Metabolic Syndrome Among Korean Adults: A Cross-Sectional Analysis of Korea National Health and Examination Survey
Source: Nutrients. 2025 May 9;17(10):1629. doi: 10.3390/nu17101629 (PMC12114246; doi:10.3390/nu17101629)
Supplement: Supplementary file 1 [file nutrients-17-01629-s001.zip › nutrients-3623688-supplementary.pdf]

Supplementary Table S1. General characteristics of the subjects aged 19-39y according to dietary monounsaturated fatty acid intake

Characteristics	Men				*p* value*	Women				*p* value*
	**Q1 (n=415)**	**Q2 (n=405)**	**Q3 (n=424)**	**Q4 (n=408)**		**Q1 (n=588)**	**Q2 (n=602)**	**Q3 (n=618)**	**Q4 (n=601)**	
Age, y	29.4 ± 0.4	29.5 ± 0.3	29.6 ± 0.3	29.1 ± 0.3	0.621	29.9 ± 0.3	29.7 ± 0.3	29.6 ± 0.3	29.2 ± 0.3	0.390
Household income[§]					0.835					0.104
Low	45 (11)	31 (10)	35 (9)	34 (9)		51 (9)	40 (8)	37 (7)	27 (6)	
Middle-low	98 (25)	100 (24)	93 (23)	92 (24)		170 (30)	150 (26)	151 (25)	144 (25)	
Middle-high	143 (36)	136 (33)	132 (33)	136 (32)		195 (32)	194 (32)	181 (31)	214 (37)	
High	116 (28)	139 (34)	142 (35)	142 (35)		162 (29)	192 (34)	211 (36)	192 (33)	
Current alcohol consumer	306 (76)	283 (69)	290 (74)	316 (77)	0.050	359 (65)	337 (60)	329 (58)	356 (63)	0.123
Current smoker	152 (37)	147 (36)	151 (37)	161 (38)	0.941	48 (9)	42 (8)	27 (5)	49 (9)	0.057
Regular aerobic exercise	230 (60)	233 (61)	232 (63)	237 (64)	0.662	289 (53)	251 (49)	273 (52)	289 (54)	0.436
Metabolic syndrome	64 (14)	74 (18)	59 (14)	62 (14)	0.428	52 (8)	48 (7)	46 (8)	29 (4)	0.034
Body mass index, kg/m^2	24.9 ± 0.2	24.8 ± 0.2	24.6 ± 0.2	24.8 ± 0.2	0.803	22.4 ± 0.2	22.1 ± 0.2	22.2 ± 0.2	22.0 ± 0.2	0.239
Waist circumference, cm	85.5 ± 0.6	85.2 ± 0.6	84.8 ± 0.5	85.6 ± 0.6	0.696	74.7 ± 0.4	73.4 ± 0.5	74.0 ± 0.5	73.5 ± 0.4	0.098
Triglyceride, mg/dL	149.6 ± 8.3	146.1 ± 5.8	145.0 ± 7.0	143.3 ± 5.3	0.929	94.8 ± 2.9	94.6 ± 5.0	91.4 ± 2.9	85.6 ± 2.3	0.068
HDL cholesterol[†], mg/dL	48.8 ± 0.6	47.9 ± 0.6	48.9 ± 0.6	48.3 ± 0.5	0.609	57.5 ± 0.6	57.3 ± 0.6	57.8 ± 0.6	59.2 ± 0.5	0.061
Fasting glucose, mg/dL	93.2 ± 0.9	95.1 ± 1.2	93.7 ± 0.7	94.4 ± 0.9	0.594	91.1 ± 0.5	89.7 ± 0.5	90.5 ± 1.0	89.4 ± 0.4	0.039
Systolic blood pressure, mmHg	116.3 ± 0.6	116.6 ± 0.6	116.5 ± 0.6	114.8 ± 0.6	0.137	106.5 ± 0.5	105.7 ± 0.5	104.9 ± 0.5	105.4 ± 0.5	0.090
Diastolic blood pressure, mmHg	78.0 ± 0.6	77.7 ± 0.5	77.6 ± 0.5	76.9 ± 0.5	0.535	71.2 ± 0.4	70.2 ± 0.4	70.5 ± 0.4	70.3 ± 0.4	0.230
Total energy intake, kcal/d	1655 ± 31	2081 ± 33	2443 ± 31	2922 ± 32	<0.001	1199 ± 21	1525 ± 22	1930 ± 24	2404 ± 28	<0.001

Carbohydrate intake, g/d	261.7 ± 5.4	309.4 ± 5.4	340.4 ± 6.0	348.9 ± 6.3	<0.001	199.8 ± 3/7	23.7 ± 4.4	275.6 ± 4.4	293.9 ± 5.0	<0.001
Fat intake, g/d	26.2 ± 0.5	44.8 ± 0.5	63.9 ± 0.7	1008. ± 1.5	<0.001	19.1 ± 0.4	34.3 ± 0.4	51.2 ± 0.5	84.4 ± 1.0	<0.001
Saturated fatty acid intake, g/d	8.8 ± 0.3	14.5 ± 0.3	20.6 ± 0.3	33.6 ± 0.6	<0.001	6.4 ± 0.2	11.4 ± 0.2	17.4 ± 0.3	28.6 ± 0.5	<0.001
Monounsaturated fatty acid intake, g/d	6.9 ± 0.1	13.4 ± 0.1	20.2 ± 0.1	35.6 ± 0.6	<0.001	5.0 ± 0.1	10.2 ± 0.1	16.0 ± 0.1	28.6 ± 0.4	<0.001
Polyunsaturated fatty acid intake, g/d	7.5 ± 0.2	12.6 ± 0.3	16.8 ± 0.4	21.5 ± 0.6	<0.001	5.6 ± 0.1	9.3 ± 0.2	12.8 ± 0.3	18.9 ± 0.5	<0.001

Data are expressed as means ± SE for continuous variables or number (%) for categorical variables.

*Differences were determined by ANOVA for continuous variables or Rao-Scott chi-square tests for categorical variables.

§For KNHANES VII-1 (2016), the cutoff values are 750.0, 1500.0, and 2463.1 thousand Korean Won. For KNHANES VII-2 (2017), the cutoff values are 894.4, 1905.7, and 3104.2 thousand Korean Won. For KNHANES VII-3 (2018), the cutoff values are 1060.7, 2020.7, and 3179.6 thousand Korean Won.

†HDL cholesterol, high-density lipoprotein cholesterol.

Supplementary Table S2. General characteristics of the subjects aged 40-64y according to dietary monounsaturated fatty acid intake

Characteristics	Men				*p* value*	Women				*p* value*
	Q1 (n=719)	Q2 (n=712)	Q3 (n=718)	Q4 (n=706)		Q1 (n=1069)	Q2 (n=1072)	Q3 (n=1075)	Q4 (n=1075)	
Age, y	53.1 ± 0.3	51.4 ± 0.3	50.6 ± 0.3	49.4 ± 0.3	<0.001	53.4 ± 0.3	51.5 ± 0.3	51.2 ± 0.2	50.0 ± 0.2	<0.001
Household income§					<0.001					<0.001
Low	129 (17)	70 (8)	44 (7)	39 (5)		183 (16)	99 (10)	88 (8)	82 (7)	
Middle-low	171 (22)	173 (19)	119 (20)	132 (18)		291 (27)	261 (24)	207 (19)	220 (21)	
Middle-high	193 (29)	263 (31)	164 (31)	215 (31)		305 (31)	310 (30)	317 (31)	319 (31)	
High	204 (32)	326 (42)	231 (41)	311 (47)		257 (26)	366 (36)	425 (42)	420 (40)	
Current alcohol consumer	482 (69)	599 (72)	411 (75)	536 (78)	0.008	385 (39)	466 (46)	447 (46)	469 (47)	0.008
Current smoker	277 (41)	334 (41)	203 (39)	256 (36)	0.221	63 (6)	47 (5)	48 (6)	36 (4)	0.643
Regular aerobic exercise	240 (39)	358 (47)	238 (46)	304 (47)	0.026	389 (42)	431 (43)	449 (46)	459 (48)	0.044
Metabolic syndrome	255 (36)	287 (35)	207 (36)	217 (29)	0.048	370 (34)	262 (25)	213 (20)	209 (20)	<0.001
Body mass index, kg/m^2	24.4 ± 0.1	24.6 ± 0.1	24.8 ± 0.1	24.7 ± 0.1	0.067	24.3 ± 0.1	23.6 ± 0.1	23.2 ± 0.1	23.4 ± 0.1	<0.001
Waist circumference, cm	86.1 ± 0.4	86.6 ± 0.3	87.0 ± 0.4	86.8 ± 0.4	0.364	80.6 ± 0.4	78.9 ± 0.3	78.0 ± 0.3	78.3 ± 0.3	<0.001
Triglyceride, mg/dL	197.1 ± 7.4	177.1 ± 5.7	181.7 ± 7.4	178.4 ± 7.6	0.189	130.1 ± 3.0	121.0 ± 3.0	116.0 ± 3.7	111.9 ± 3.3	<0.001
HDL cholesterol†, mg/dL	45.7 ± 0.5	46.9 ± 0.5	46.7 ± 0.5	47.0 ± 0.5	0.240	53.0 ± 0.5	54.2 ± 0.4	56.6 ± 0.5	55.9 ± 0.5	<0.001
Fasting glucose, mg/dL	107.5 ± 1.1	106.3 ± 1.0	106.9 ± 1.4	102.4 ± 0.7	<0.001	102.4 ± 0.9	98.2 ± 0.7	97.0 ± 0.6	97.4 ± 0.6	<0.001
Systolic blood pressure, mmHg	122.4 ± 0.7	120.4 ± 0.6	121.5 ± 0.7	119.3 ± 0.6	0.002	119.4 ± 0.6	115.8 ± 0.5	114.8 ± 0.6	114.0 ± 0.5	<0.001
Diastolic blood pressure, mmHg	80.8 ± 0.5	80.9 ± 0.4	81.7 ± 0.5	80.9 ± 0.4	0.361	76.8 ± 0.3	75.8 ± 0.3	75.5 ± 0.3	75.2 ± 0.3	0.005
Total energy intake, kcal/d	1703 ± 26	2098 ± 25	2401 ± 27	2855 ± 24	<0.001	1244 ± 16	1558 ± 17	1737 ± 18	2159 ± 22	<0.001

Carbohydrate intake, g/d	284.1 ± 4.5	325.8 ± 4.2	350.4 ± 5.1	361.0 ± 5.1	<0.001	233.7 ± 3.4	269.5 ± 3.7	274.9 ± 3.6	297.8 ± 4.0	<0.001
Fat intake, g/d	18.3 ± 0.3	34.4 ± 0.3	50.3 ± 0.4	84.7 ± 1.1	<0.001	14.1 ± 0.2	26.3 ± 0.2	38.2 ± 0.3	66.0 ± 0.8	<0.001
Saturated fatty acid intake, g/d	6.1 ± 0.1	10.9 ± 0.2	15.7 ± 0.2	27.2 ± 0.4	<0.001	4.6 ± 0.1	8.3 ± 0.1	11.8 ± 0.1	21.3 ± 0.4	<0.001
Monounsaturated fatty acid intake, g/d	4.4 ± 0.1	9.9 ± 0.1	15.5 ± 0.1	29.7 ± 0.5	<0.001	3.5 ± 0.1	7.5 ± 0.0	11.9 ± 0.1	22.7 ± 0.3	<0.001
Polyunsaturated fatty acid intake, g/d	5.8 ± 0.1	10.3 ± 0.2	14.3 ± 0.3	19.6 ± 0.4	<0.001	4.6 ± 0.1	8.1 ± 0.1	10.9 ± 0.2	16.0 ± 0.3	<0.001

Data are expressed as means ± SE for continuous variables or number (%) for categorical variables.

*Differences were determined by ANOVA for continuous variables or Rao-Scott chi-square tests for categorical variables.

§For KNHANES VII-1 (2016), the cutoff values are 750.0, 1500.0, and 2463.1 thousand Korean Won. For KNHANES VII-2 (2017), the cutoff values are 894.4, 1905.7, and 3104.2 thousand Korean Won. For KNHANES VII-3 (2018), the cutoff values are 1060.7, 2020.7, and 3179.6 thousand Korean Won.

†HDL cholesterol, high-density lipoprotein cholesterol.

Supplementary Table S3. General characteristics of the subjects aged ≥65y according to dietary monounsaturated fatty acid intake

Characteristics	Men				*p* value*	Women				*p* value*
	**Q1 (n=454)**	**Q2 (n=454)**	**Q3 (n=458)**	**Q4 (n=460)**		**Q1 (n=598)**	**Q2 (n=583)**	**Q3 (n=602)**	**Q4 (n=606)**	
Age, y	73.7 ± 0.3	72.9 ± 0.3	72.1 ± 0.3	71.8 ± 0.3	<0.001	74.5 ± 0.2	73.4 ± 0.3	72.4 ± 0.2	71.4 ± 0.2	<0.001
Household income§					<0.001					<0.001
Low	251 (57)	201 (46)	126 (26)	119 (29)		380 (65)	320 (56)	263 (42)	214 (40)	
Middle-low	113 (25)	124 (28)	129 (30)	138 (32)		110 (21)	127 (24)	161 (28)	156 (25)	
Middle-high	39 (11)	59 (16)	108 (26)	92 (21)		40 (9)	64 (13)	84 (19)	97 (17)	
High	22 (7)	41 (10)	66 (19)	77 (19)		23 (5)	40 (8)	49 (10)	85 (18)	
Current alcohol consumer	221 (53)	233 (55)	245 (61)	272 (63)	0.045	67 (12)	105 (20)	95 (18)	123 (22)	0.002
Current smoker	82 (19)	77 (17)	67 (16)	71 (18)	0.710	12 (3)	11 (2)	16 (3)	9 (1)	0.346
Regular aerobic exercise	114 (31)	152 (38)	157 (40)	160 (42)	0.032	107 (22)	147 (30)	149 (28)	180 (33)	0.013
Metabolic syndrome	161 (37)	149 (34)	172 (37)	156 (38)	0.746	342 (62)	325 (58)	290 (50)	278 (49)	<0.001
Body mass index, kg/m^2	23.5 ± 0.2	23.5 ± 0.2	23.8 ± 0.2	24.0 ± 0.1	0.145	24.8 ± 0.2	24.4 ± 0.1	24.3 ± 0.2	24.4 ± 0.2	0.133
Waist circumference, cm	86.3 ± 0.5	85.9 ± 0.5	86.9 ± 0.5	87.7 ± 0.5	0.050	85.1 ± 0.5	84.2 ± 0.5	83.1 ± 0.4	84.2 ± 0.4	0.029
Triglyceride, mg/dL	131.8 ± 3.9	129.8 ± 4.2	131.1 ± 3.8	131.1 ± 3.9	0.989	143.9 ± 5.8	140.5 ± 5.2	133.6 ± 5.2	127.1 ± 3.1	0.021
HDL cholesterol†, mg/dL	45.5 ± 0.6	46.2 ± 0.6	45.8 ± 0.7	47.1 ± 0.6	0.234	47.8 ± 0.7	49.2 ± 0.6	49.9 ± 0.6	50.8 ± 0.5	0.003
Fasting glucose, mg/dL	109.8 ± 1.6	109.0 ± 1.4	110.2 ± 1.5	109.7 ± 1.5	0.952	107.8 ± 1.4	108.3 ± 1.4	106.3 ± 1.2	105.9 ± 1.4	0.567
Systolic blood pressure, mmHg	127.1 ± 0.9	127.8 ± 1.0	124.6 ± 0.8	126.6 ± 0.9	0.057	131.4 ± 1.1	130.6 ± 0.9	128.9 ± 0.9	129.5 ± 1.0	0.261
Diastolic blood pressure, mmHg	71.7 ± 0.6	71.8 ± 0.6	71.5 ± 0.5	73.4 ± 0.6	0.057	72.3 ± 0.5	71.9 ± 0.5	72.4 ± 0.5	73.1 ± 0.5	0.377
Total energy intake, kcal/d	1439 ± 29	1738 ± 32	2014 ± 35	2417 ± 34	<0.001	1101 ± 17	1304 ± 21	1522 ± 23	1899 ± 30	<0.001

Carbohydrate intake, g/d	273.6 ± 5.7	308.0 ± 6.2	338.0 ± 7.1	353.1 ± 5.8	<0.001	226.6 ± 3.9	250.8 ± 4.7	274.5 ± 5.0	299.0 ± 5.9	<0.001
Fat intake, g/d	10.8 ± 0.4	20.6 ± 0.3	32.2 ± 0.4	59.7 ± 1.2	<0.001	6.8 ± 0.2	14.3 ± 0.2	23.4 ± 0.3	47.0 ± 1.0	<0.001
Saturated fatty acid intake, g/d	3.7 ± 0.2	6.4 ± 0.2	9.3 ± 0.2	18.2 ± 0.4	<0.001	2.1 ± 0.1	4.5 ± 0.1	7.1 ± 0.2	14.3 ± 0.4	<0.001
Monounsaturated fatty acid intake, g/d	2.1 ± 0.1	5.3 ± 0.1	9.3 ± 0.1	20.2 ± 0.5	<0.001	1.4 ± 0.0	3.7 ± 0.0	6.7 ± 0.1	15.5 ± 0.3	<0.001
Polyunsaturated fatty acid intake, g/d	3.8 ± 0.1	6.8 ± 0.2	10.6 ± 0.3	15.5 ± 0.4	<0.001	2.6 ± 0.1	4.8 ± 0.1	7.4 ± 0.2	12.8 ± 0.4	<0.001

Data are expressed as means ± SE for continuous variables or number (%) for categorical variables.

*Differences were determined by ANOVA for continuous variables or Rao-Scott chi-square tests for categorical variables.

[§]For KNHANES VII-1 (2016), the cutoff values are 750.0, 1500.0, and 2463.1 thousand Korean Won. For KNHANES VII-2 (2017), the cutoff values are 894.4, 1905.7, and 3104.2 thousand Korean Won. For KNHANES VII-3 (2018), the cutoff values are 1060.7, 2020.7, and 3179.6 thousand Korean Won.

[†]HDL cholesterol, high-density lipoprotein cholesterol.

Supplementary Table S4. Risks of abdominal obesity[1] according to quartiles of dietary monounsaturated fatty acid intake among Korean adults by age and sex

Monounsaturated	Unadjusted		Model 1*		Model 2†		Model 3‡	
fatty acid intake	OR	95% CI	OR	95% CI	OR	95% CI	OR	95% CI
**19-39y**								
Men								
Q1	1 (ref)		1 (ref)		1 (ref)		1 (ref)	
Q2	0.97	0.69, 1.34	1.15	0.62, 2.15	1.00	0.52, 1.93	1.02	0.53, 1.95
Q3	1.00	0.73, 1.39	1.47	0.74, 2.91	1.55	0.77, 3.11	1.62	0.80, 3.28
Q4	1.04	0.76, 1.44	1.81	0.89, 3.70	1.78	0.83, 3.81	1.93	0.84, 4.39
Women								
Q1	1 (ref)		1 (ref)		1 (ref)		1 (ref)	
Q2	0.78	0.58, 1.06	0.65	0.38, 1.12	0.65	0.37, 1.13	0.66	0.37, 1.21
Q3	0.89	0.67, 1.19	0.62	0.35, 1.09	0.60	0.33, 1.08	0.63	0.32, 1.25
Q4	0.78	0.58, 1.05	0.52	0.25, 1.08	0.56	0.27, 1.17	0.61	0.24, 1.56
**40-64y**								
Men								
Q1	1 (ref)		1 (ref)		1 (ref)		1 (ref)	
Q2	1.30	0.99, 1.69	1.45	0.98, 2.13	1.56	1.05, 2.32	1.47	0.98, 2.21
Q3	1.39	1.05, 1.84	1.17	0.77, 1.78	1.29	0.82, 2.03	1.16	0.74, 1.83
Q4	1.33	1.01, 1.74	1.23	0.78, 1.95	1.39	0.86, 2.25	1.14	0.68, 1.91
Women								
Q1	1 (ref)		1 (ref)		1 (ref)		1 (ref)	
Q2	0.69	0.55, 0.85	0.95	0.69, 1.32	0.94	0.67, 1.32	0.91	0.64, 1.29
Q3	0.59	0.48, 0.72	1.04	0.76, 1.44	1.10	0.78, 1.55	1.03	0.69, 1.55
Q4	0.64	0.52, 0.78	0.88	0.62, 1.26	0.94	0.65, 1.36	0.85	0.52, 1.39
**≥65y**								
Men								
Q1	1 (ref)		1 (ref)		1 (ref)		1 (ref)	
Q2	0.74	0.52, 1.05	0.72	0.41, 1.28	0.74	0.41, 1.34	0.75	0.41, 1.38
Q3	0.89	0.62, 1.27	0.83	0.47, 1.47	0.89	0.49, 1.63	0.92	0.49, 1.73
Q4	1.12	0.81, 1.54	1.17	0.68, 2.02	1.34	0.75, 2.39	1.43	0.70, 2.90
Women								
Q1	1 (ref)		1 (ref)		1 (ref)		1 (ref)	
Q2	0.81	0.59, 1.11	0.83	0.53, 1.28	0.87	0.55, 1.37	0.94	0.58, 1.55

Q3	0.65	0.47, 0.89	0.60	0.39, 0.93	0.57	0.36, 0.91	0.68	0.40, 1.15
Q4	0.82	0.61, 1.12	0.98	0.59, 1.62	0.93	0.55, 1.55	1.27	0.56, 2.87

[1]Abdominal obesity was defined as waist circumference ≥90 cm in men or ≥80 cm in women.

* Model 1: adjusted for age, body mass index, and total energy intake

† Model 2: model 1 plus household income, alcohol consumption, smoking, and aerobic exercise

‡ Model 3: model 2 plus energy from carbohydrates

Supplementary Table S5. Risks of hypo-HDL-cholesterolemia[1] according to quartiles of dietary monounsaturated fatty acid intake among Korean adults by age and sex

Monounsaturated	Unadjusted		Model 1*		Model 2†		Model 3‡	
fatty acid intake	OR	95% CI	OR	95% CI	OR	95% CI	OR	95% CI
**19-39y**								
Men								
Q1	1 (ref)		1 (ref)		1 (ref)		1 (ref)	
Q2	1.07	0.73, 1.55	1.03	0.69, 1.54	1.03	0.68, 1.57	1.07	0.70, 1.64
Q3	0.87	0.60, 1.26	0.83	0.54, 1.27	0.90	0.58, 1.39	0.96	0.61, 1.51
Q4	0.88	0.62, 1.25	0.76	0.49, 1.20	0.77	0.48, 1.25	0.88	0.52, 1.48
Women								
Q1	1 (ref)		1 (ref)		1 (ref)		1 (ref)	
Q2	1.08	0.81, 1.44	1.31	0.96, 1.77	1.31	0.95, 1.80	1.35	0.96, 1.89
Q3	1.07	0.79, 1.45	1.48	1.03, 2.13	1.38	0.94, 2.03	1.46	0.93, 2.27
Q4	0.75	0.55, 1.02	1.27	0.83, 1.94	1.31	0.84, 2.04	1.43	0.80, 2.55
**40-64y**								
Men								
Q1	1 (ref)		1 (ref)		1 (ref)		1 (ref)	
Q2	0.79	0.61, 1.03	0.85	0.64, 1.11	0.91	0.67, 1.23	0.96	0.71, 1.30
Q3	0.76	0.58, 1.01	0.85	0.63, 1.16	0.92	0.66, 1.29	1.02	0.72, 1.43
Q4	0.76	0.58, 0.98	0.96	0.69, 1.35	1.01	0.71, 1.44	1.20	0.82, 1.76
Women								
Q1	1 (ref)		1 (ref)		1 (ref)		1 (ref)	
Q2	0.77	0.62, 0.96	0.83	0.66, 1.04	0.85	0.67, 1.08	0.93	0.73, 1.20
Q3	0.64	0.52, 0.79	0.71	0.56, 0.90	0.72	0.56, 0.93	0.86	0.66, 1.14
Q4	0.69	0.56, 0.84	0.71	0.55, 0.92	0.74	0.57, 0.97	1.00	0.71, 1.40
**≥65y**								
Men								
Q1	1 (ref)		1 (ref)		1 (ref)		1 (ref)	
Q2	0.87	0.63, 1.20	0.95	0.68, 1.34	1.09	0.77, 1.55	1.17	0.82, 1.68
Q3	1.01	0.74, 1.38	1.16	0.82, 1.64	1.30	0.91, 1.84	1.46	0.99, 2.16
Q4	0.76	0.54, 1.08	0.99	0.66, 1.48	1.05	0.70, 1.59	1.32	0.81, 2.15
Women								
Q1	1 (ref)		1 (ref)		1 (ref)		1 (ref)	
Q2	0.94	0.71, 1.23	0.94	0.71, 1.26	0.95	0.70, 1.28	0.92	0.67, 1.28

Q3	0.77	0.58, 1.02	0.76	0.55, 1.04	0.79	0.57, 1.10	0.75	0.51, 1.11
Q4	0.69	0.53, 0.92	0.63	0.45, 0.88	0.67	0.47, 0.96	0.62	0.38, 0.99

[1]Hypo-HDL-cholesterolemia was defined as HDL-cholesterol <40 mg/dL in men or <50 mg/dL in women.

* Model 1: adjusted for age, body mass index, and total energy intake

† Model 2: model 1 plus household income, alcohol consumption, smoking, and aerobic exercise

‡ Model 3: model 2 plus energy from carbohydrates

Supplementary Table S6. Risks of hypertension[1] according to quartiles of dietary monounsaturated fatty acid intake among Korean adults by age and sex

Monounsaturated	Unadjusted		Model 1*		Model 2†		Model 3‡	
fatty acid intake	OR	95% CI	OR	95% CI	OR	95% CI	OR	95% CI
**19-39y**								
Men								
Q1	1 (ref)		1 (ref)		1 (ref)		1 (ref)	
Q2	1.38	0.85, 2.22	1.46	0.84, 2.53	1.36	0.78, 2.39	1.37	0.77, 2.43
Q3	1.28	0.79, 2.07	1.39	0.79, 2.46	1.25	0.69, 2.29	1.26	0.67, 2.36
Q4	0.93	0.54, 1.60	1.02	0.48, 2.17	0.90	0.41, 1.98	0.91	0.37, 2.23
Women								
Q1	1 (ref)		1 (ref)		1 (ref)		1 (ref)	
Q2	0.77	0.35, 1.68	0.64	0.25, 1.64	0.65	0.24, 1.74	0.62	0.20, 1.91
Q3	0.52	0.19, 1.41	0.43	0.14, 1.33	0.44	0.14, 1.43	0.42	0.10, 1.80
Q4	0.47	0.19, 1.11	0.37	0.11, 1.26	0.37	0.10, 1.34	0.34	0.06, 1.95
**40-64y**								
Men								
Q1	1 (ref)		1 (ref)		1 (ref)		1 (ref)	
Q2	1.00	0.79, 1.27	1.08	0.83, 1.40	1.09	0.83, 1.42	1.02	0.77, 1.34
Q3	1.02	0.80, 1.31	1.12	0.83, 1.50	1.10	0.81, 1.49	0.98	0.72, 1.34
1.26Q4	0.71	0.58, 0.96	0.88	0.64, 1.22	0.93	0.66, 1.31	0.77	0.53, 1.10
Women								
Q1	1 (ref)		1 (ref)		1 (ref)		1 (ref)	
Q2	0.75	0.59, 0.94	0.96	0.75, 1.24	0.97	0.75, 1.26	0.98	0.75, 1.30
Q3	0.67	0.52, 0.84	0.94	0.73, 1.22	0.96	0.73, 1.25	0.98	0.71, 1.36
Q4	0.54	0.42, 0.70	0.80	0.59, 1.10	0.82	0.60, 1.14	0.86	0.56, 1.33
**≥65y**								
Men								
Q1	1 (ref)		1 (ref)		1 (ref)		1 (ref)	
Q2	0.88	0.64, 1.21	0.95	0.68, 1.34	1.06	0.75, 1.50	1.00	0.70, 1.43
Q3	0.66	0.48, 0.90	0.70	0.50, 0.99	0.74	0.51, 1.08	0.67	0.45, 1.00
Q4	0.72	0.53, 0.99	0.77	0.52, 1.14	0.74	0.49, 1.12	0.61	0.38, 0.98
Women								
Q1	1 (ref)		1 (ref)		1 (ref)		1 (ref)	
Q2	0.80	0.60, 1.07	0.89	0.66, 1.21	0.88	0.64, 1.21	0.87	0.61, 1.22

Q3	0.69	0.52, 0.92	0.85	0.62, 1.16	0.90	0.65, 1.26	0.88	0.58, 1.33
Q4	0.64	0.48, 0.86	0.81	0.56, 1.16	0.86	0.59, 1.25	0.82	0.47, 1.45

[1]Hypertension was defined as blood pressure ≥130/85 mmHg or usage of blood pressure medication.

* Model 1: adjusted for age, body mass index, and total energy intake

† Model 2: model 1 plus household income, alcohol consumption, smoking, and aerobic exercise

‡ Model 3: model 2 plus energy from carbohydrates

Supplementary Table S7. Risks of hyperglycemia[1] according to quartiles of dietary monounsaturated fatty acid intake among Korean adults by age and sex

Monounsaturated fatty acid intake	Unadjusted		Model 1*		Model 2†		Model 3‡	
	OR	95% CI	OR	95% CI	OR	95% CI	OR	95% CI
**19-39y**								
Men								
Q1	1 (ref)		1 (ref)		1 (ref)		1 (ref)	
Q2	1.30	0.90, 1.87	1.30	0.87, 1.95	1.30	0.85, 1.99	1.32	0.86, 2.03
Q3	1.16	0.79, 1.70	1.16	0.74, 1.84	1.12	0.71, 1.78	1.16	0.72, 1.87
Q4	1.27	0.88, 1.82	1.28	0.78, 2.09	1.23	0.73, 2.09	1.32	0.75, 2.32
Women								
Q1	1 (ref)		1 (ref)		1 (ref)		1 (ref)	
Q2	0.94	0.62, 1.42	0.94	0.60, 1.49	0.95	0.59, 1.54	0.97	0.58, 1.61
Q3	0.84	0.54, 1.30	0.80	0.48, 1.32	0.81	0.48, 1.36	0.83	0.46, 1.51
Q4	0.74	0.49, 1.12	0.72	0.40, 1.27	0.68	0.39, 1.21	0.72	0.35, 1.47
**40-64y**								
Men								
Q1	1 (ref)		1 (ref)		1 (ref)		1 (ref)	
Q2	0.85	0.68, 1.07	0.85	0.67, 1.09	0.94	0.73, 1.22	0.85	0.66, 1.10
Q3	0.99	0.75, 1.30	0.99	0.74, 1.32	1.09	0.81, 1.48	0.92	0.68, 1.26
Q4	0.78	0.61, 0.98	0.81	0.61, 1.08	0.88	0.65, 1.19	0.65	0.47, 0.90
Women								
Q1	1 (ref)		1 (ref)		1 (ref)		1 (ref)	
Q2	0.71	0.58, 0.87	0.85	0.69, 1.06	0.85	0.68, 1.07	0.80	0.63, 1.01
Q3	0.57	0.46, 0.71	0.74	0.58, 0.95	0.74	0.58, 0.96	0.66	0.50, 0.88
Q4	0.65	0.52, 0.81	0.88	0.66, 1.16	0.87	0.65, 1.16	0.72	0.50, 1.03
**≥65y**								
Men								
Q1	1 (ref)		1 (ref)		1 (ref)		1 (ref)	
Q2	1.24	0.90, 1.70	1.32	0.95, 1.83	1.35	0.96, 1.89	1.27	0.89, 1.79
Q3	1.24	0.93, 1.65	1.37	1.01, 1.85	1.59	1.14, 2.21	1.42	1.00, 2.02
Q4	1.22	0.90, 1.66	1.44	1.04, 2.06	1.52	1.05, 2.20	1.23	0.80, 1.91
Women								
Q1	1 (ref)		1 (ref)		1 (ref)		1 (ref)	
Q2	0.99	0.75, 1.31	1.16	0.87, 1.55	1.19	0.87, 1.62	1.17	0.86, 1.61

Q3	1.04	0.78, 1.39	1.40	1.04, 1.88	1.39	1.03, 1.87	1.35	0.97, 1.89
Q4	0.93	0.68, 1.27	1.44	1.01, 2.05	1.48	1.03, 2.12	1.41	0.86, 2.32

[1]Hyperglycemia was defined as fasting glucose ≥100 mg/dL, usage of glucose lowering medication, or insulin treatment.

* Model 1: adjusted for age, body mass index, and total energy intake

† Model 2: model 1 plus household income, alcohol consumption, smoking, and aerobic exercise

‡ Model 3: model 2 plus energy from carbohydrates
